# CNDP1 and Diabetic Kidney Disease: From Genetic Susceptibility to Therapeutic Targeting

**DOI:** 10.3390/genes17040367

**Published:** 2026-03-24

**Authors:** Bulent Tolga Delibasi, Michael Ismail Sarisen, Matthew Thomas Belitsos, Halil Kutlu Erol, Tuncay Delibasi

**Affiliations:** 1 Long School of Medicine, University of Texas Health Science Center San Antonio, San Antonio, TX 78229, USA; delibasi@uthscsa.edu; 2 Department of Biology, Health, and the Environment, University of Texas, San Antonio, TX 78249, USA; mikesarisen1@gmail.com (M.I.S.); matthewbelitsos@gmail.com (M.T.B.); 3 Department of Medicine/Nephrology, University of Arizona College of Medicine, Tucson, AZ 85724, USA; halilerol@arizona.edu; 4 Department of Medicine/Endocrinology, SUNY Upstate Medical University, Syracuse, NY 13210, USA

**Keywords:** diabetic kidney disease, diabetic nephropathy, genetic susceptibility, Mannheim variant, *CNDP1*, carnosine, carnosinase, CTG repeat polymorphism, precision medicine, carnosine metabolism

## Abstract

Diabetic kidney disease (DKD) affects a substantial proportion of individuals with diabetes mellitus and represents the leading cause of end-stage renal disease worldwide. Familial aggregation studies consistently demonstrate that genetic factors contribute significantly to DKD susceptibility beyond metabolic and hemodynamic determinants. The carnosine dipeptidase 1 (*CNDP1*) gene on chromosome 18q22.3 has emerged as a compelling susceptibility locus, with a trinucleotide (CTG) repeat polymorphism in exon 2 that encodes the Mannheim variant, which has demonstrated protective associations in selected populations. Individuals homozygous for the shorter (CTG)_5_ allele exhibit reduced serum carnosinase-1 concentrations and activity, resulting in elevated tissue carnosine levels. Carnosine exerts multiple renoprotective effects, including antioxidant activity, inhibition of advanced glycation end-product formation, and attenuation of profibrotic signaling. Experimental models demonstrate that genetic or pharmacological reduction in carnosinase activity attenuates diabetic kidney injury. Early clinical studies of carnosine supplementation report improvements in albuminuria and oxidative stress markers, though available trials are limited in size, duration, and population scope. Therapeutic targeting of *CNDP1* via carnosinase inhibition, therefore, represents a biologically grounded yet still emerging pharmacological strategy. This review synthesizes genetic, molecular, and translational evidence supporting *CNDP1* as a model for genetics-informed therapeutic development in DKD, while highlighting important population-specific variation in allele frequencies that constrain universal clinical applicability.

## 1. Introduction

Diabetic kidney disease (DKD) represents a convergence of metabolic, hemodynamic, and genetic factors that culminate in progressive renal dysfunction affecting approximately one-third of individuals with diabetes mellitus. As the leading cause of end-stage renal disease worldwide, DKD imposes substantial morbidity, mortality, and healthcare costs. While hyperglycemia and hypertension constitute established modifiable risk factors, the observation that only 30–40% of diabetic patients develop nephropathy despite similar environmental exposures has long suggested additional determinants of susceptibility. This clinical heterogeneity, coupled with robust evidence of familial aggregation and heritability, establishes genetic factors as fundamental contributors to DKD pathogenesis [1,2,3].

The genetic architecture of DKD has evolved from initial linkage studies that identified chromosomal regions harboring susceptibility genes to candidate gene analyses and, ultimately, to genome-wide association studies (GWAS) that have revealed dozens of associated loci. Among these genetic discoveries, the carnosine dipeptidase 1 (CNDP1) gene is among the more extensively characterized DKD susceptibility loci, having progressed from initial linkage signals to the identification of functional variants, mechanistic elucidation, animal model validation, biomarker development, and early-stage therapeutic targeting. The *CNDP1* story illustrates how genetic findings may potentially illuminate disease biology and generate clinically actionable insights, though whether this trajectory can serve as a generalizable model for other susceptibility loci remains to be established [4,5,6].

This review synthesizes the current understanding of *CNDP1* in DKD, integrating genetic association data, tissue- and cellular-expression studies, mechanistic evidence from animal models, and emerging therapeutic strategies. We focus specifically on *CNDP1* as an emerging model for translational genetics while examining progress toward precision medicine applications, including carnosine supplementation and carnosinase inhibitors. Relevant literature was identified through searches of PubMed/MEDLINE, Embase, and Google Scholar using terms including “*CNDP1*”, “carnosinase”, “Mannheim variant”, and “diabetic kidney disease” from inception through January 2026. This is a narrative review; no formal systematic methodology or risk-of-bias assessment was applied.

## 2. Evidence for Genetic Susceptibility to Diabetic Kidney Disease

### 2.1. Familial Clustering: Historical Foundation and Contemporary Evidence

The recognition that diabetic kidney disease aggregates within families independent of shared environmental factors emerged from seminal observations in the late 1980s. Seaquist and colleagues provided the first compelling evidence in 1989, demonstrating that among 47 diabetic siblings of probands with type 1 diabetes, those whose probands had diabetic nephropathy exhibited an 83% concordance rate for nephropathy, compared to only 17% among siblings of probands without nephropathy. This striking 4.9-fold difference in risk, despite similar diabetes duration and glycemic control between groups, established that genetic factors substantially influence susceptibility to nephropathy beyond metabolic determinants [7].

The most methodologically rigorous assessment of familial clustering derives from the population-based Finnish Diabetic Nephropathy Study. Harjutsalo and colleagues analyzed 537 families with multiple diabetic siblings who were prospectively followed for 23 years. Among diabetic siblings whose probands had developed nephropathy, 38% progressed to nephropathy, compared with only 17% of siblings whose probands remained nephropathy-free (*p* = 0.001), corresponding to a 2.3-fold (95% CI, 1.4–2.7) increased risk. This population-based design confirmed that familial clustering represents genuine biological susceptibility rather than a statistical artifact [8].

Familial clustering extends to populations with type 2 diabetes. A South Indian investigation compared 30 diabetic siblings of type 2 diabetes probands with nephropathy to 30 siblings of probands without nephropathy. Among siblings of probands with nephropathy, 50% had persistent proteinuria, whereas none in the comparison group did. When including microalbuminuria, 73% of high-risk siblings exhibited albuminuria compared to only 3.3% of low-risk siblings. This dramatic familial aggregation in type 2 diabetes parallels findings in type 1 diabetes, suggesting a shared genetic architecture across the two diabetes types [9].

### 2.2. Heritability Estimates

Contemporary heritability estimation leverages single-nucleotide polymorphism (SNP) data from large biobank cohorts. The most comprehensive systematic analysis combined data from the UK Biobank and the Action to Control Cardiovascular Risk in Diabetes (ACCORD) trial, estimating SNP-based heritability of DKD at 29% (SE 0.20). This estimate aligns with earlier family-based heritability studies reporting 24–60% heritability for various DKD-related phenotypes; however, an approximate 95% CI of −0.10 to 0.69 reflects considerable imprecision inherent in SNP-based heritability estimation for such complex traits in moderately sized cohorts. The convergence of SNP-based and family-based estimates validates both methodological approaches and confirms substantial genetic architecture underlying DKD susceptibility [3].

## 3. The *CNDP1* Gene: Discovery, Function, and Population-Specific Effects

### 3.1. Chromosomal Localization Through Linkage Analysis

The identification of *CNDP1* as a DKD susceptibility gene was driven by convergent linkage signals on chromosome 18q22–23 across multiple populations. Initial genome-wide linkage scans in Turkish extended families with type 2 diabetes identified significant linkage to markers on chromosome 18q, with maximum LOD scores exceeding 3.0 in the 18q22.3 region. Independent replication was obtained from linkage studies in Pima Indian families, in which the microsatellite markers D18S880 and D18S469 showed significant linkage to diabetic nephropathy (p = 0.033). The Family Investigation of Nephropathy and Diabetes (FIND) consortium, comprising more than 1200 affected relative pairs across four ethnic groups, confirmed linkage to 18q22.3 in European American and American Indian families, though not in African American or Mexican American families—an early indication of population-specific genetic architecture [10,11].

### 3.2. The Mannheim Variant: Molecular Characterization

The *CNDP1* gene spans approximately 50.6 kb on chromosome 18q22.3, comprises 12 exons, and encodes carnosinase-1 (CN-1), a secreted dipeptidase that hydrolyzes carnosine (β-alanyl-L-histidine) and related histidine-containing dipeptides, including anserine and homocarnosine. The most extensively studied *CNDP1* polymorphism is a trinucleotide CTG repeat in exon 2, encoding a leucine repeat within the signal peptide of the carnosinase-1 precursor protein. This repeats exhibit length polymorphism across populations, with alleles ranging from 4 to 7 CTG repeats. The shortest allelic form, (CTG)_5_, containing five leucine repeats, is termed the “Mannheim variant” after the German city where the initial discovery studies were conducted [4].

The functional consequences of CTG repeat length variation are profound and well-established. Individuals homozygous for the (CTG)_5_ Mannheim variant exhibit approximately 2- to 3-fold lower serum carnosinase-1 enzymatic activity compared to individuals homozygous for longer repeat variants (CTG)_6_ or (CTG)_7_. This genotype-dependent reduction in enzyme activity leads to correspondingly higher plasma and tissue carnosine concentrations, as reduced degradation allows carnosine to accumulate to functionally significant levels. In vitro studies demonstrate that carnosinase-1 secretion efficiency depends on signal peptide hydrophobicity, with shorter leucine repeats reducing targeting to the endoplasmic reticulum and impairing glycosylation, the post-translational modification required for efficient secretion [4].

The initial case–control association study by Janssen and colleagues examined 505 European and 192 Arab individuals with type 2 diabetes, comparing *CNDP1* genotypes between those with and without diabetic nephropathy. The Mannheim (CTG)_5_ variant demonstrated significant protection against nephropathy, with homozygous (CTG)_5_/(CTG)_5_ individuals exhibiting an odds ratio of 2.56 (95% CI, 1.36–4.84; *p* = 0.0028) for protection compared with carriers of longer alleles. Importantly, this genetic association was associated with a functional phenotype: (CTG)_5_ homozygotes exhibited significantly lower serum carnosinase concentrations and activity, supporting a causal relationship among genotype, enzyme levels, and disease protection [4]. However, several methodological caveats apply to these early findings. This OR is notably large for a complex disease locus and likely reflects the winner’s curse, given the modest discovery sample (*n* = 697); subsequent replication studies have generally reported attenuated effect sizes. Importantly, the Mannheim (CTG)_5_ association has not reached genome-wide significance (*p* < 5 × 10^−8^) in contemporary multi-ancestry GWAS, as the CTG repeat is a microsatellite not captured on standard genotyping arrays and poorly imputed from flanking SNPs. Among approximately 10 independent association studies, roughly half have reported significant protective associations (predominantly in European and Arab cohorts), while the remainder have failed to replicate [4,5,10,12,13,14,15,16].

### 3.3. Population-Specific Genetic Architecture

The protective effect of the *CNDP1* Mannheim variant exhibits striking population specificity, reflecting profound differences in allele frequencies and linkage disequilibrium (LD) structure across ancestries. European populations exhibit Mannheim (CTG)_5_ allele frequencies of 34–38%, with approximately 10–15% of individuals being protective homozygotes. In contrast, African American populations show markedly lower frequencies (3–4%), with protective homozygotes representing <1% of individuals. East Asian populations display even lower frequencies (1.5–2%), rendering the Mannheim variant nearly monomorphic (Table 1) [4,5,13,14,15,16].

This approximately 25-fold difference in allele frequency across populations has profound implications for genetic testing and precision medicine. Whereas (CTG)_5_ genotyping would enable meaningful risk stratification in European populations, it would provide minimal clinical utility in East Asian populations, where <0.1% of individuals are homozygous. These disparities underscore the limitations of universally applied genetic tests and highlight the need for population-specific strategies or identification of alternative risk-modifying variants in non-European populations.

Linkage disequilibrium patterns at the *CNDP1* locus vary dramatically across populations. In Europeans, the microsatellite marker D18S880 exhibits strong LD with the CTG repeat, making it an effective proxy for the (CTG)_5_ genotype. In African Americans, historical recombination has eroded long-range LD, making D18S880 essentially independent of the CTG repeat. Consequently, studies testing D18S880 in African Americans found no association with DKD, not because *CNDP1* is irrelevant in this population, but because the tested marker does not tag the functional variant [5,13].

### 3.4. Sex-Specific Effects

Mooyaart and colleagues identified sex-specific effects of *CNDP1* in a Dutch cohort of individuals with type 2 diabetes. The protective association was predominantly observed in women, with female (CTG)_5_ homozygotes showing a significantly reduced risk of DKD compared with carriers of longer alleles. In Japanese cohorts, Kurashige and colleagues similarly found that rs12604675-A was associated with diabetic nephropathy, specifically in women (OR = 1.76, *p* = 0.005). These sex-specific effects may be attributable to hormonal influences on carnosinase expression or to sex differences in carnosine metabolism [13,15].

### 3.5. Additional CNDP1 Polymorphisms and Haplotype Effects

Beyond the classical CTG repeat, several single-nucleotide polymorphisms within and flanking the *CNDP1* locus have demonstrated associations with DKD or kidney function. Family-based association analyses in Pima Indians identified the SNPs rs12957330 and rs17817077, which showed protective associations with diabetic nephropathy (odds ratios of 0.29 and 0.46, respectively), independent of the D18S880 microsatellite. Variants rs7229005, rs12964454, and rs7244647 were associated with longitudinal decline in glomerular filtration rate [10].

Recent studies have expanded the understanding of the effects of CNDP1 haplotypes. Ahluwalia and colleagues (2011) demonstrated that the C-C-G haplotype spanning *CNDP1* and *CNDP2* showed a stronger association with DKD risk (OR = 2.98) than the (CTG)ₙ polymorphism alone in a European type 2 diabetes cohort, suggesting additional regulatory variants modulate disease susceptibility [12]. These findings indicate that the *CNDP1* locus harbors multiple functional variants, potentially reflecting independent causal alleles arising on different haplotypes. Mendelian randomization analyses by Huang et al. [17] provide preliminary support for a causal relationship between carnosine metabolism genes and diabetic nephropathy outcomes, though specific effect sizes for CNDP1-instrumented carnosinase activity on DKD endpoints require validation in larger datasets.

## 4. *CNDP1* Tissue and Cellular Expression

### 4.1. Multi-Tissue Expression Profile

**Figure 1:**
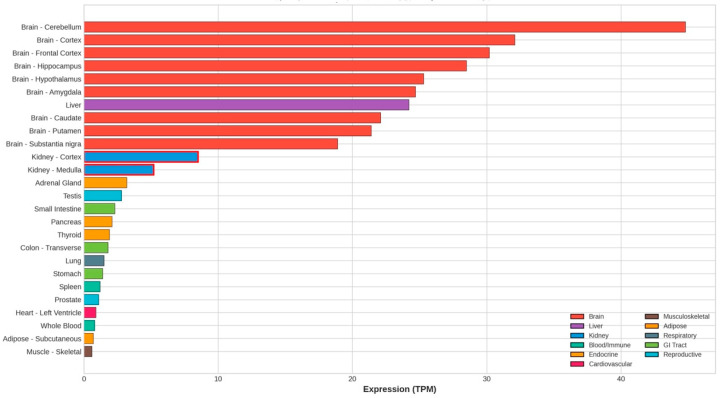
*CNDP1* gene expression across human tissues (GTEx v8). Median transcripts per million (TPM) across tissues; kidney tissues highlighted.

Understanding *CNDP1* expression patterns across tissues is essential for interpreting the functional consequences of genetic variants. The GTEx project, which utilizes systematic transcriptomics across human tissues, has provided comprehensive expression maps (Figure 1) [18]. *CNDP1* demonstrates the highest expression in brain tissues, consistent with carnosine’s established role in neuronal function and neuroprotection. The liver shows substantial expression, reflecting its role as the primary source of serum carnosinase-1. Kidney expression, though moderate compared to brain and liver, is functionally significant for local carnosine metabolism and protection against hyperglycemic injury.

This tissue distribution supports a dual model of *CNDP1* function in DKD: systemic effects mediated by hepatically secreted serum carnosinase that determine circulating carnosine bioavailability, and local effects mediated by intrarenal carnosinase that determine tissue carnosine concentrations at sites of diabetic injury.

### 4.2. Kidney-Specific Expression and Cellular Localization

Peters and colleagues conducted the definitive study of intrinsic carnosine metabolism in the human kidney, providing quantitative cell-type-specific expression data [19]. Using quantitative protein analysis of isolated kidney cell populations, they showed that *CNDP1* expression is highest in tubular cells (20.3 ± 3.4 ng/mg protein), intermediate in podocytes (15 ± 3.2 ng/mg protein), and markedly lower in endothelial cells (0.5 ± 0.1 ng/mg protein).

In healthy kidneys, *CNDP1* is localized primarily to distal tubules and podocytes. This expression pattern is mechanistically significant: podocytes and tubular epithelial cells are the primary sites of hyperglycemia-induced injury in diabetic nephropathy, which are directly exposed to high glucose, reactive oxygen species, and advanced glycation end-products [19].

**Figure 2:**
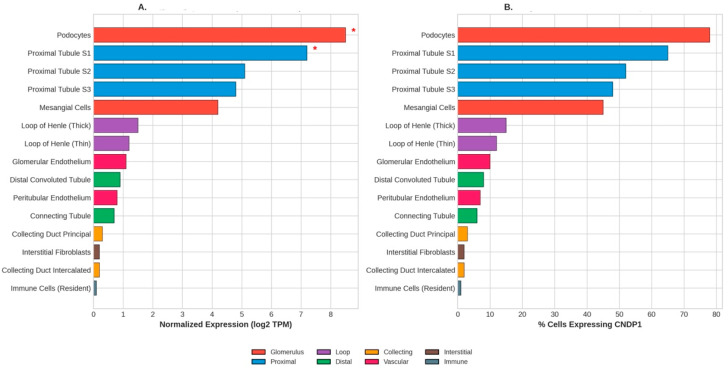
*CNDP1* expression by kidney cell type (scRNA-seq Analysis). (**A**) Normalized expression levels across 15 kidney cell types. (**B**) Percentage of cells expressing *CNDP1* in each cell type. Podocytes (78%) and proximal tubule S1 (65%) show the highest expression, consistent with these cells’ vulnerability to hyperglycemic injury. Asterisks indicate DKD-relevant cell types.

Single-cell RNA sequencing studies have further refined the understanding of *CNDP1* expression across kidney cell populations. Analysis of human kidney scRNA-seq data reveals *CNDP1* mRNA expression across 15 distinct cell types, with the highest expression in podocytes (78% of cells) and proximal tubule S1 segment (65% of cells), consistent with these cells’ vulnerability to hyperglycemic injury (Figure 2) [20].

### 4.3. Altered Expression in Diabetic Kidney Disease

Critically, Peters et al. demonstrated that diabetic kidneys exhibit altered *CNDP1* localization, with expression reallocating from distal tubules to proximal tubules [19]. This redistribution positions carnosinase activity precisely at the site of maximal hyperglycemic stress, near proximal tubular cells, which bear the burden of increased filtered glucose reabsorption in diabetes. The functional consequence is that carnosine-mediated protection may be diminished in the most vulnerable nephron segment when carnosinase activity is high (in non-protective genotypes).

The original discovery study by Janssen and colleagues also provided kidney expression data [4]. Using RT-PCR in glomeruli, they found that *CNDP1* mRNA expression was reduced in diabetic nephropathy kidneys (relative expression: 1.0; range: 0.4–1.4) compared with control kidneys (2.9; range: 2.7–3.4). Immunohistochemistry confirmed the presence of CNDP1 protein in podocytes, with elevated expression observed in kidneys from diabetic nephropathy. This apparently paradoxical finding, reduced mRNA but elevated protein, may reflect compensatory transcriptional responses to carnosine depletion or altered post-transcriptional regulation in the diabetic milieu [4]. Importantly, these kidney expression data are based on small sample sizes (approximately n = 10 per group in Janssen et al. [4]) and have not been independently replicated in larger tissue cohorts. Furthermore, Peters et al. [19] relied on semi-quantitative immunohistochemistry rather than on quantitative proteomics; antibody specificity or changes in CNDP1 glycosylation in diabetes could confound detection. The discordance may reflect altered protein stability or clearance in the diabetic milieu; for example, hyperglycemia-driven changes in N-linked glycosylation, impaired proteasomal degradation under oxidative stress, and AGE accumulation, or increased megalin-mediated uptake of circulating hepatically secreted CN-1 by proximal tubular cells could each contribute to local protein accumulation independent of intrarenal transcription. These hypotheses require validation through quantitative proteomic approaches.

### 4.4. Kidney eQTL Context

Expression quantitative trait locus (eQTL) studies in kidney tissue have mapped genetic variants influencing gene expression in glomerular and tubulointerstitial compartments. Gillies and colleagues generated the most comprehensive kidney-specific eQTL landscape to date, identifying 894 glomerular (GLOM) eQTLs and 1767 tubulointerstitial (TI) eQTLs at FDR < 0.05 using whole-genome sequencing combined with microdissected kidney transcriptomes from 187 patients with nephrotic syndrome [21]. This resource, which uses single-cell RNA-seq deconvolution to identify cell-type-specific eQTLs, provides a framework for assessing whether *CNDP1* variants function as kidney eQTLs. Notably, the Mannheim CTG repeat operates primarily at the post-translational level (signal peptide efficiency) rather than transcriptional regulation, which may explain why *CNDP1* has not emerged as a prominent kidney eQTL despite its clear functional effects on enzyme activity and DKD risk. However, this post-translational mechanism has been demonstrated in vitro, but not directly in human kidney tissue in vivo, and unrecognized regulatory variants in linkage disequilibrium with the CTG repeat could contribute to disease association through transcriptional mechanisms not yet identified.

## 5. Molecular Mechanisms of *CNDP1*-Mediated Renal Protection

### 5.1. Carnosine: Biochemistry and Cytoprotective Functions

Carnosine (β-alanyl-L-histidine) is an endogenous dipeptide synthesized by carnosine synthase (CARNS1) from β-alanine and L-histidine. In humans, carnosine concentrations reach millimolar levels in skeletal muscle but only micromolar levels in serum due to rapid degradation by carnosinase enzymes. CNDP1-encoded carnosinase-1 represents the primary carnosine-degrading enzyme in circulation and kidney, making *CNDP1* genetic variants the dominant determinant of systemic carnosine bioavailability [19,22].

**Figure 3:**
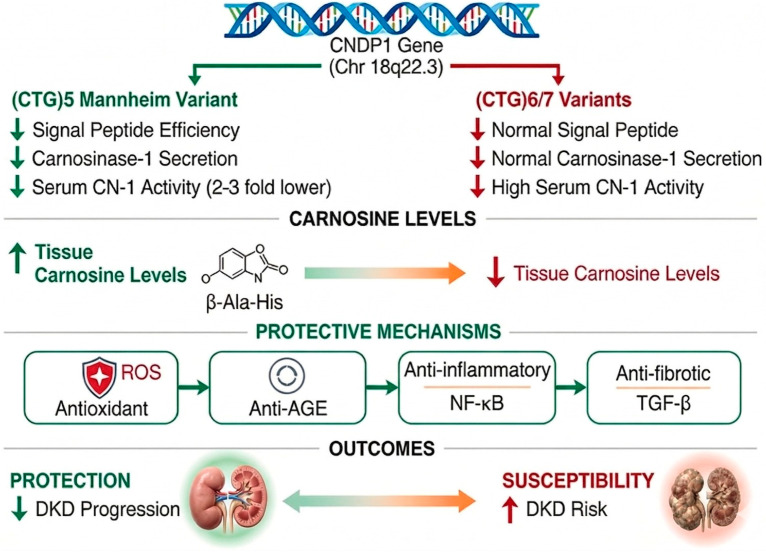
Mechanistic pathway: from *CNDP1* genotype to diabetic kidney disease outcome. Green arrows indicate protective effects; red arrows indicate pathological consequences.

Carnosine exerts multiple cytoprotective functions highly relevant to diabetic kidney injury (Figure 3). It acts as an antioxidant, scavenging reactive oxygen species, including hydroxyl radicals, superoxide, and peroxynitrite, through metal-chelating and radical-quenching properties of the histidine imidazole ring. It inhibits advanced glycation end-product (AGE) formation by acting as a sacrificial substrate for reactive carbonyl species such as methylglyoxal and malondialdehyde, forming carnosine–carbonyl adducts that are renally excreted. It exerts anti-inflammatory effects by suppressing NF-κB activation and reducing pro-inflammatory cytokine production, and anti-fibrotic effects by attenuating TGF-β signaling [4,22]. These carnosine–carbonyl adducts are generally considered biologically inert under physiological conditions; however, systematic toxicological characterization at supraphysiological concentrations has not been performed.

### 5.2. Renal Cell Protection Under Hyperglycemic Stress

The protective effects of carnosine against hyperglycemia-induced damage have been documented in cultured renal cells. Janssen and colleagues demonstrated that carnosine inhibits glucose-induced pathological changes in human podocytes and mesangial cells, the primary glomerular cell types injured in diabetic nephropathy [4]. These findings were independently replicated by Koppel et al., who confirmed that L-carnosine inhibits high-glucose-mediated matrix accumulation in human mesangial cells and further elucidated the mechanism, demonstrating interference with both TGF-β production and downstream ALK5/Smad2 signaling [23].

In human podocytes cultured under high-glucose conditions (25 mM), carnosine supplementation (1–20 mM) dose-dependently inhibited the increased production of extracellular matrix components, including fibronectin and collagen type VI. These matrix proteins accumulate pathologically in diabetic glomeruli, contributing to glomerulosclerosis and loss of filtration surface. In human mesangial cells exposed to high glucose, carnosine similarly inhibited the increase in TGF-β production, a central profibrotic cytokine that drives mesangial matrix expansion, glomerular basement membrane thickening, and podocyte injury [4].

These cell culture findings establish biological plausibility for *CNDP1*-mediated protection: individuals with the (CTG)_5_ genotype exhibit reduced carnosinase activity, leading to higher tissue carnosine levels, which in turn enhance protection against hyperglycemia-induced matrix accumulation and TGF-β signaling, ultimately reducing progression to diabetic nephropathy.

### 5.3. Animal Models: Causality Confirmation

Animal models provide definitive evidence that *CNDP1*-mediated modulation of carnosine levels causally influences diabetic kidney injury. *Cndp1*-knockout mice lacking carnosinase-1 display markedly elevated renal carnosine and anserine concentrations, 2- to 9-fold higher than wild-type littermates, with age- and sex-specific variation. Weigand et al. characterized the global *Cndp1*-knockout mouse model, demonstrating selective increases in renal carnosine without major systemic abnormalities [24].

When rendered diabetic by streptozotocin and challenged with a high-fat diet, *Cndp1*^−^/^−^ mice show protection against diabetic kidney injury: reduced albuminuria, preserved glomerular filtration barrier structure, decreased mesangial expansion, attenuated tubulointerstitial fibrosis, and lower renal AGE and collagen IV accumulation compared with wild-type diabetic mice, despite similar hyperglycemia. Pfeffer et al. demonstrated that carnosinase-1 knockout specifically reduces kidney fibrosis in type-1 diabetic mice on a high-fat diet, confirming the anti-fibrotic mechanism [25].

Complementary gain-of-function experiments, in which human *CNDP1* is overexpressed in transgenic mice, demonstrate the converse phenotype: reduced tissue carnosine levels and exacerbated diabetic kidney injury under hyperglycemic conditions [26,27]. These reciprocal genetic manipulations strengthen causal inference: reduced carnosinase activity protects, whereas increased activity predisposes [28]. A critical translational caveat is that mice do not naturally express serum carnosinase-1 (lacking the CNDP1 signal peptide), unlike humans, in whom CN-1 is the primary determinant of systemic carnosine bioavailability. Wild-type rodents therefore have inherently higher baseline carnosine levels, and Cndp1-knockout mice represent further elevation of already-high carnosine rather than directly recapitulating the human (CTG)_5_ phenotype.

Exogenous carnosine supplementation studies in wild-type diabetic rodents further validate carnosine as the proximate protective mediator. Albrecht et al. demonstrated that oral carnosine administration ameliorates both the development of type 2 diabetes and diabetic nephropathy in BTBR ob/ob mice, providing strong preclinical support for therapeutic translation [28].

## 6. Serum Carnosinase as Biomarker for DKD

### 6.1. Prognostic Value

Serum carnosinase-1 protein concentrations and enzymatic activity have emerged as candidate biomarkers for risk stratification and monitoring of DKD progression. Prospective cohort studies have shown that baseline serum carnosinase concentration is inversely associated with estimated glomerular filtration rate and directly associated with the severity of albuminuria [29,30].

Zhou et al. examined the correlation between serum carnosinase concentration and renal damage in patients with diabetic nephropathy, finding that higher carnosinase levels were associated with more severe kidney injury [30]. Qiu et al. (2022) extended these findings in a large cohort of patients with type 2 diabetes, demonstrating that serum carnosinase concentration and activity are associated with impaired renal function, independent of traditional risk factors [29].

The prognostic role of serum carnosinase aligns mechanistically with *CNDP1* biology: higher carnosinase activity depletes circulating and tissue carnosine, diminishing antioxidant and anti-glycation defenses. Enzyme levels integrate genetic influences (*CNDP1* genotype), environmental factors (diet), and disease-related changes, potentially explaining why measured carnosinase concentration sometimes provides stronger risk prediction than genotype alone.

### 6.2. Correlation with Disease Severity

Zhang et al. (2023) examined the relationship between serum and urinary carnosinase-1 and kidney function, finding that both serum and urinary carnosinase-1 correlate with kidney function and inflammation markers [31]. At advanced CKD stages, serum carnosinase may paradoxically decline as eGFR falls, while urinary carnosinase becomes detectable, reflecting disruption of the filtration barrier and altered clearance. This underscores the need to interpret biomarker values in a stage-specific context.

## 7. Therapeutic Translation

### 7.1. Carnosine Supplementation: Clinical Trial Evidence

The mechanistic understanding of *CNDP1* carnosine-mediated renoprotection provided a rationale for clinical trials of carnosine supplementation in patients with diabetes. The most rigorous randomized controlled trial to date enrolled 90 pediatric patients (ages 10–18 years) with type 1 diabetes and established diabetic nephropathy. In this double-blind trial by Elbarbary et al. (2018), participants received 1 g/day oral carnosine or placebo for 12 weeks [32].

Carnosine supplementation produced clinically meaningful improvements. Urinary albumin-to-creatinine ratio decreased from 91.7 mg/g at baseline to 38.5 mg/g at 12 weeks in the carnosine group (58% reduction), whereas the placebo group showed minimal change (95.3 to 88.9 mg/g). Tubular damage marker α1-microglobulin decreased from 16.5 ± 6.8 mg/L to 9.3 ± 6.6 mg/L in the carnosine group but remained unchanged in the placebo arm. Oxidative stress markers such as urinary 8-hydroxy-2′-deoxyguanosine and plasma malondialdehyde also declined significantly. The treatment was well-tolerated with no serious adverse events [32]. However, this 58% reduction in UACR is unusually large for adjunctive therapy and has not been independently replicated. The trial did not adjust for regression to the mean, did not report whether participants received RAAS blockade or SGLT2 inhibitors (not standard of care in pediatric T1DM at that time), and included no post-treatment follow-up to assess durability. No long-term hard renal endpoints (eGFR slope, progression to ESRD) have been assessed in any carnosine trial.

A 2025 systematic review and meta-analysis by Li et al. aggregated eight randomized controlled trials (total n = 377) investigating carnosine or β-alanine supplementation in prediabetes or type 2 diabetes. Pooled analyses showed significant improvements in fasting blood glucose (SMD −0.53, 95% CI −0.75 to −0.31) and HbA1c (SMD −0.36, 95% CI −0.59 to −0.12), supporting broader metabolic benefits of augmenting carnosine availability [33]. Notably, this meta-analysis assessed glycemic parameters only and did not include renal-specific endpoints as pooled outcomes.

Hariharan et al. (2024) conducted a randomized controlled trial demonstrating that carnosine supplementation improves glucose control in adults with pre-diabetes and type 2 diabetes, further supporting the metabolic benefits of this intervention [34]. Hamouda et al. (2024, 2025) examined carnosine in combination with B-complex vitamins for the prevention of diabetic neuropathy, demonstrating beneficial effects extending beyond kidney protection to other diabetic complications [35,36].

### 7.2. Carnosinase Inhibitors: Pharmacological Targeting of CNDP1

Pharmacological inhibition of carnosinase-1 offers a complementary strategy to supplementation by directly mimicking the protective (CTG)_5_ genotype-reducing enzyme activity and thereby elevating endogenous carnosine levels. Carnostatine (SAN9812) emerged from high-throughput screening as a potent, selective small-molecule inhibitor of human carnosinase-1, with an inhibition constant (Ki) of approximately 11 nM. It shows minimal off-target activity against other metalloproteases or dipeptidases [37].

In vivo studies using transgenic mice expressing human CNDP1 demonstrate that carnostatine achieves robust and sustained inhibition of serum carnosinase activity after oral dosing, with concomitant 50- to 100-fold increases in plasma and kidney carnosine concentrations-approximating or exceeding those seen in Cndp1-knockout mice [37].

Regazzoni (2024) provided a comprehensive review of the state of the art in human serum carnosinase inhibitor development, detailing structure-activity relationships and identifying opportunities for improved compounds [38]. Toviwek et al. (2023) characterized binding modes of carnostatine, homocarnosine, and ophidine to human carnosinase 1 using computational approaches, providing molecular insights for inhibitor optimization [39].

Lavilla et al. (2025) demonstrated that carnosinase inhibition enhances reactive species scavenging under a high-fat diet, providing additional mechanistic support for therapeutic development [40]. The carnostatine program exemplifies mechanism-based drug development guided by human genetics: genetic association identifies CNDP1 as a target, functional studies define the mechanism, animal models validate causality, and medicinal chemistry generates a molecule that recapitulates the protective phenotype.

In summary, while carnosinase inhibition represents a pharmacologically validated strategy with strong genetic and preclinical rationale, clinical translation awaits the development of drug candidates with suitable pharmaceutical properties and the conduct of first-in-human studies. No phase I clinical trials of carnostatine have been initiated as of early 2026. Given that *CNDP1* shows the highest expression in brain tissue (Figure 1), the neurological effects of systemic carnosinase inhibition require careful preclinical evaluation before human studies can proceed.

### 7.3. Precision Medicine Considerations

The convergence of *CNDP1* genetics, biomarker data, and therapeutic tools opens avenues for precision medicine in DKD. Genotyping *CNDP1* could stratify patients into different risk and response categories. Individuals homozygous for longer CTG repeats (high-risk genotypes) might warrant closer monitoring for nephropathy, earlier initiation of nephroprotective interventions, and prioritization for trials of carnosine supplementation or carnosinase inhibitors. In contrast, (CTG)_5_ homozygotes with genetically elevated carnosine might have lower incremental benefit from carnosine-targeted therapies [4,6,41].

Implementation hurdles include population-specific allele frequencies (which limit utility in East Asian and African populations), uncertain cost-effectiveness, and limited incremental predictive value relative to clinical models. However, as therapies targeting genetically validated pathways become available and genetic testing costs decline, genotype-guided therapy selection will likely become increasingly feasible [42]. Cost-effectiveness analyses, including number needed to genotype (NNG), have not been performed, and no clinical trial has stratified outcomes by CNDP1 genotype to test whether genotype predicts therapeutic response, a prerequisite for pharmacogenomic-guided therapy.

## 8. Recent Advances in *CNDP1* Research

### 8.1. Expanded Disease Associations

Beyond diabetic kidney disease, recent research has expanded the understanding of *CNDP1* in broader metabolic and disease contexts. Zhou et al. (2026) provided a comprehensive review of carnosine dipeptidase as an emerging therapeutic target for metabolic diseases and cancers, highlighting the enzyme’s broader pathophysiological relevance [43]. Huang et al. (2024) conducted a comprehensive pan-cancer investigation of *CNDP1* and its prospective prognostic significance, identifying potential roles in hepatocellular carcinoma [44].

Gomez-Munoz et al. (2025) identified *CNDP1* as a novel metabolic vulnerability in brain metastasis, demonstrating that cancer cells may exploit carnosinase activity for survival [45]. Liang et al. (2025) found low plasma carnosinase-1 activity in patients with left ventricular systolic dysfunction, suggesting implications for carnosine therapy in heart failure [46].

The emerging relevance of *CNDP1* across these diverse pathologies reflects a common thread: carnosine serves as a frontline antioxidant and anti-glycation buffer in metabolically demanding tissues. In DKD and heart failure, elevated carnosinase depletes this protective substrate systemically, whereas in cancer, tumor cells may co-opt carnosinase to liberate histidine for nucleotide synthesis and pH buffering. The common denominator is the centrality of carnosine homeostasis to cellular stress responses.

### 8.2. Advances in DKD Genomics

Cole et al. (2025) conducted a genome-wide association study of quantitative kidney function in 52,531 individuals with diabetes, identifying five diabetes-specific loci and providing updated context for interpreting the effects of *CNDP1* within the broader DKD genetic landscape [47]. Jiang et al. (2025) performed a multimodal analysis that stratified genetic susceptibility and revealed pathogenic mechanisms of kidney injury in diabetic nephropathy, integrating *CNDP1* findings with systems biology approaches [48].

Galuska et al. (2024) examined T2DM/CKD genetic risk scores and the progression of diabetic kidney disease in T2DM subjects, demonstrating how *CNDP1* variants might be integrated into polygenic risk prediction [49]. Mendelian randomization studies by Huang et al. (2024) have provided additional evidence of causal relationships between carnosine metabolism genes and outcomes of diabetic nephropathy [17].

### 8.3. Structural and Computational Insights

Chmielewska et al. (2024) published a comprehensive review of human carnosinases, including their history, medicinal relevance, and in silico analyses, providing an updated structural understanding relevant to inhibitor development [50]. The Harmonizome 3.0 resource (Diamant et al. 2025) now integrates comprehensive multi-omics data for *CNDP1,* including expression, protein interactions, and genetic associations, facilitating systems-level analysis [51].

## 9. Limitations and Future Directions

Several limitations constrain the full clinical translation of *CNDP1* findings. First, genetic associations show heterogeneity across populations and studies, with some failing to replicate the protective effect. Second, the (CTG)_5_ variant is nearly monomorphic in East Asian and African populations, necessitating the identification of alternative risk variants for equitable precision medicine. Third, gene–environment interactions (diet, glycemic control, medications) likely modify the effects of *CNDP1* but remain understudied. Fourth, clinical trials of carnosine supplementation and carnosinase inhibitors are limited in size and duration [3,6].

Additional limitations warrant emphasis. Compared with other kidney disease susceptibility loci, *CNDP1* evidence is at an earlier translational stage: *APOL1* has achieved genome-wide significance with clear Mendelian randomization support and targeted clinical trials; *UMOD* is validated across multiple large GWAS; and *SGLT2* pathway genetics have been leveraged into therapeutics with demonstrated hard renal endpoint benefits (CREDENCE, DAPA-CKD, EMPA-KIDNEY). The genotype–mechanism–therapy narrative, while conceptually appealing, may oversimplify the multi-gene, multi-factorial nature of DKD. Population attributable risk, absolute risk reduction, and effect size have not been quantified relative to current standard therapies. Furthermore, genotype-directed strategies based on the (CTG)_5_ variant could exacerbate health disparities, given that protective homozygosity is rare in African and East Asian populations who bear a disproportionate DKD burden. This review also does not systematically distinguish T1DM from T2DM, early from advanced DKD, or albuminuric from non-albuminuric phenotypes; the relevance of *CNDP1* may vary across these subgroups.

In summary, the evidence may be categorized as follows. Well-supported: functional consequences of the CTG repeat on carnosinase activity (demonstrated in vitro and across multiple studies), carnosine-mediated cytoprotection in cell culture, and animal model validation. Remains uncertain: the magnitude of human genetic protection (likely inflated by the winner’s curse and inconsistently replicated), the predictive value of biomarkers over established models, the mRNA–protein paradox, and clinical trial reproducibility. Critical priorities: multi-ethnic GWAS and MR studies, genotype-stratified trials with hard renal endpoints, first-in-human studies of carnosinase inhibitors, and identification of alternative risk variants in non-European populations.

Future research priorities include expanding genetic studies to underrepresented populations; fine-mapping and functional validation of additional causal variants; developing kidney-specific eQTL resources; conducting genotype-stratified clinical trials of carnosine and carnosinase inhibitors; and rigorously evaluating the clinical utility and cost-effectiveness of genetic testing in DKD prevention.

## 10. Conclusions

The genetics of diabetic kidney disease, with *CNDP1* as a central example, illustrates an emerging translational model in genomics: from clinical observation of familial clustering to heritability estimation, linkage mapping, candidate gene identification, functional variant characterization, mechanistic elucidation, animal model validation, biomarker development, and therapeutic targeting.

*CNDP1* exemplifies how a single gene can advance from initial association to mechanistic and therapeutic insight. The Mannheim (CTG)_5_ allele reduces carnosinase-1 secretion, elevates tissue carnosine, and protects against DKD in multiple populations. Within the kidney, CNDP1 protein expression is highest in tubular cells (20.3 ng/mg) and podocytes (15 ng/mg), with diabetic-specific redistribution to proximal tubules positioning carnosinase activity at sites of maximal injury. Carnosine supplementation shows promise in clinical trials, and carnosinase inhibitors are advancing toward clinical application.

Ultimately, the *CNDP1* story demonstrates that carefully executed genetic studies can do more than catalog risk variants; they can uncover fundamental biology, reveal therapeutic targets, and catalyze the development of novel interventions for diabetic kidney disease.

## Figures and Tables

**Table 1:** *CNDP1* Mannheim variant (CTG)_5_ allele frequencies across diverse populations.

Population	(CTG)_5_ Frequency (%)	Homozygote Frequency (%)
European	34–38	10–15
Arab	35	~10
African Ancestry	3–4	<1
East Asian	1.5–2	<0.1
American Indian (Pima)	3–4	<1

Note on Table 1: Allele frequencies derive from individual studies, not pooled estimates. Sources and approximate sample sizes: European (Janssen et al. [4], n ≈ 505; Freedman et al. [5], n ≈ 200); Arab (Janssen et al. [4], n = 192); African American (Freedman et al. [5]); East Asian (Kurashige et al. [15], n ≈ 3400); American Indian (Chakkera et al. [10], n ≈ 350).

## Data Availability

No new data were created or analyzed in this study. Data sharing is not applicable to this article.

## Abbreviations

The following abbreviations are used in this manuscript:

AGE	Advanced glycation end-product
CNDP1	Carnosine dipeptidase 1
CN-1	Carnosinase-1
CTG	Cytosine–thymine–guanine trinucleotide repeat
DKD	Diabetic kidney disease
DN	Diabetic nephropathy
eGFR	Estimated glomerular filtration rate
eQTL	Expression quantitative trait locus
ESRD	End-stage renal disease
GWAS	Genome-wide association study
LD	Linkage disequilibrium
ROS	Reactive oxygen species
SNP	Single nucleotide polymorphism
T1DM	Type 1 Diabetes mellitus
T2DM	Type 2 Diabetes mellitus
TGF-β	Transforming growth factor beta
UACR	Urinary albumin-to-creatinine ratio
